# 2-(3,4-Di­meth­oxy­phen­yl)-1-pentyl-4,5-diphenyl-1*H*-imidazole

**DOI:** 10.1107/S1600536813031759

**Published:** 2013-11-27

**Authors:** Shaaban K. Mohamed, Mehmet Akkurt, Adel A. Marzouk, Kuldip Singh, Mustafa R. Albayati

**Affiliations:** a Chemistry and Environmental Division, Manchester Metropolitan University, Manchester M1 5GD, England; b Chemistry Department, Faculty of Science, Minia University, 61519 El-Minia, Egypt; c Department of Physics, Faculty of Sciences, Erciyes University, 38039 Kayseri, Turkey; d Pharmaceutical Chemistry Department, Faculty of Pharmacy, Al Azhar University, Egypt; e Department of Chemistry, University of Leicester, Leicester, England; f Kirkuk University, College of Science, Department of Chemistry, Kirkuk, Iraq

## Abstract

The central imidazole ring in the title compound, C_28_H_30_N_2_O_2_, makes dihedral angles of 28.42 (13), 71.22 (15) and 29.50 (14)°, respectively, with the phenyl rings in the 4- and 5-positions and the 3,4-di­meth­oxy­phenyl group. In the crystal, mol­ecules are linked by C—H⋯O and C—H⋯N hydrogen bonds, weak π–π stacking inter­actions [centroid–centroid distance = 3.760 (2) Å] and C—H⋯π contacts, forming a three-dimensional network.

## Related literature

For medicinal and industrial applications of imidazole-containing compounds see: Oyeka & Gugnani (1992 ▶); Schrekker *et al.* (2013 ▶); Mital (2009 ▶); Juchau (1989 ▶); Rondu *et al.* (1997 ▶); Bousquet & Feldman (1999 ▶); Ueno *et al.* (1995 ▶); Jung & Huang (2000 ▶); Isobe *et al.* (2001 ▶). For similar structures, see: Akkurt *et al.* (2013 ▶); Mohamed *et al.* (2013 ▶).
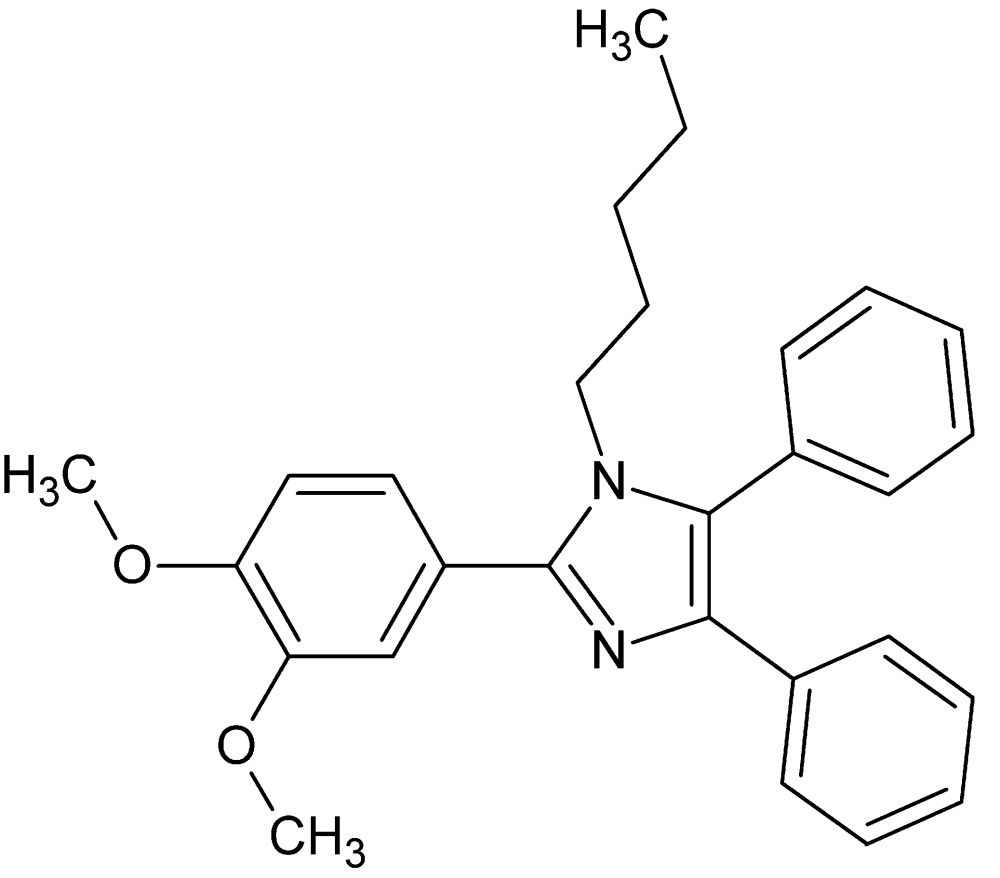


## Experimental

### 

#### Crystal data

C_28_H_30_N_2_O_2_
*M* *_r_* = 426.54
Triclinic, 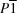

*a* = 8.900 (2) Å
*b* = 11.915 (3) Å
*c* = 12.009 (3) Å
α = 106.757 (6)°
β = 96.007 (5)°
γ = 108.170 (6)°
*V* = 1131.8 (5) Å^3^
*Z* = 2
Mo *K*α radiation
μ = 0.08 mm^−1^
*T* = 150 K
0.24 × 0.21 × 0.11 mm

#### Data collection

Bruker APEX2000 CCD area-detector diffractometer
Absorption correction: multi-scan (*SADABS*; Bruker, 2011 ▶) *T*_min_ = 0.668, *T*_max_ = 0.981
8350 measured reflections
3953 independent reflections
2219 reflections with *I* > 2σ(*I*)
*R*_int_ = 0.086

#### Refinement

*R*[*F* ^2^ > 2σ(*F* ^2^)] = 0.058
*wR*(*F* ^2^) = 0.127
*S* = 0.87
3953 reflections
292 parameters
H-atom parameters constrained
Δρ_max_ = 0.25 e Å^−3^
Δρ_min_ = −0.25 e Å^−3^

### 

Data collection: *SMART* (Bruker, 2011 ▶); cell refinement: *SAINT* (Bruker, 2011 ▶); data reduction: *SAINT*; program(s) used to solve structure: *SHELXS97* (Sheldrick, 2008 ▶); program(s) used to refine structure: *SHELXL97* (Sheldrick, 2008 ▶); molecular graphics: *ORTEP-3 for Windows* (Farrugia, 2012 ▶); software used to prepare material for publication: *WinGX* (Farrugia, 2012 ▶) and *PLATON* (Spek, 2009 ▶).

## Supplementary Material

Crystal structure: contains datablock(s) global, I. DOI: 10.1107/S1600536813031759/sj5371sup1.cif

Structure factors: contains datablock(s) I. DOI: 10.1107/S1600536813031759/sj5371Isup2.hkl

Supplementary material file. DOI: 10.1107/S1600536813031759/sj5371Isup3.cml

Additional supplementary materials:  crystallographic information; 3D view; checkCIF report

## Figures and Tables

**Table 1:** Hydrogen-bond geometry (Å, °) *Cg*1 is the centroid of the N1/N2/C1–C3 imidazole ring.

*D*—H⋯*A*	*D*—H	H⋯*A*	*D*⋯*A*	*D*—H⋯*A*
C23—H23⋯O1^i^	0.95	2.53	3.168 (4)	125
C27—H27*A*⋯N2^ii^	0.98	2.51	3.474 (3)	168
C27—H27*A*⋯*Cg*1^ii^	0.98	2.77	3.608 (3)	144

Symmetry codes: (i) 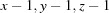; (ii) 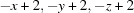.

**Fig. 1.**
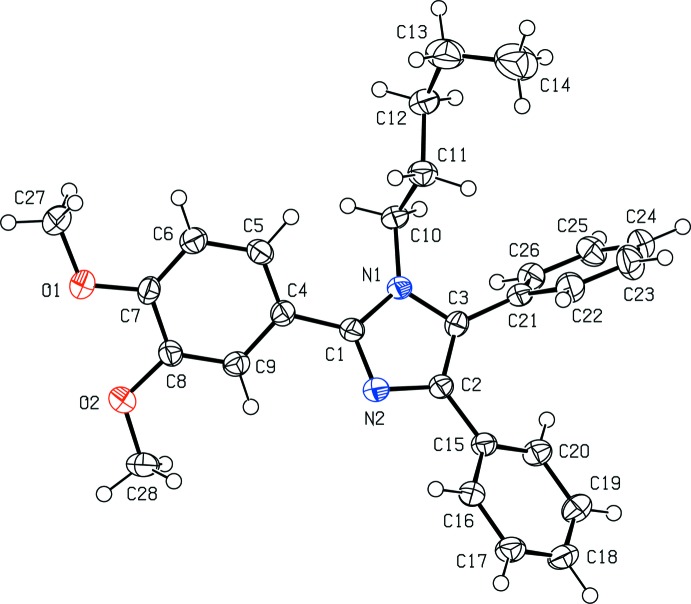
The molecular structure of the title compound with the atom numbering scheme. Displacement ellipsoids for non-H atoms are drawn at the 50% probability level.

**Fig. 2.**
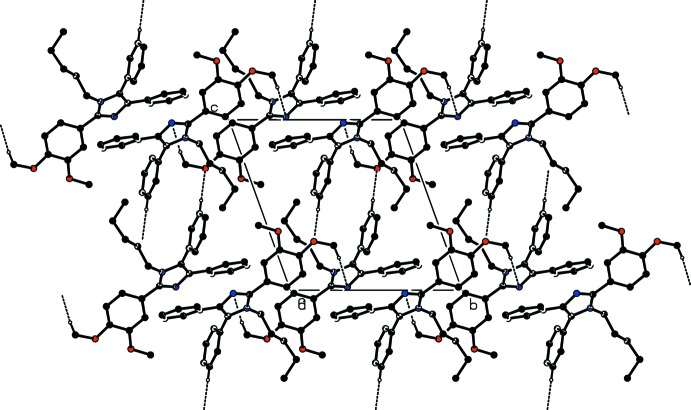
Crystal packing of the title compound viewed along the a axis. Hydrogen bonds are drawn as dashed lines. H atoms not involved in hydrogen bonding have been omitted for clarity.

## supplementary crystallographic information

### 1. Comment

Many drugs contain an imidazole ring. Examples include butoconazole, clomidazole, clotrimazole, croconazole, econazole, fenticonazole, ketoconazole, isoconazole miconazole, neticonazole, omoconazole, oxiconazole, sertaconazole, sulconazole and tioconazole all of which act as antifungal drugs (Oyeka & Gugnani, 1992, Schrekker *et al.*, 2013). Antibiotic drugs such as metronidazole, tinidazole and nimorazole also contain imidazoles (Mital, 2009; Juchau, 1989). Moreover, 2-substituted imidazolines are used as synthetic intermediates (Rondu *et al.*, 1997), catalysts (Bousquet & Feldman, 1999), chiral auxiliaries (Ueno *et al.*, 1995), chiral catalysts (Jung & Huang, 2000) and ligands for asymmetric catalysis (Isobe *et al.*, 2001)in various synthetic reactions. Based on such facts, and as an extension of our work concerning the production bio-active heterocyclic compounds, we report in this study the synthesis and crystal structure of another imidazole derivative.

In the non-planar title compound (I), (Fig. 1), the dihedral angles between the (N1/N2/C1–C3) imidazole ring, and the two phenyl rings (C15–C20 and C21–C26) and the 3,4-dimethoxyphenyl group (C4–C9) are 28.42 (13), 71.22 (15) and 29.50 (14)°, respectively.

The N1–C10–C11–C12 and C11–C12–C13–C14 torsion angles are 173.6 (2) and 66.4 (4)°, respectively. The bond lengths and bond angles in (I) are normal and comparable to those reported for the similar compounds (Akkurt *et al.*, 2013; Mohamed *et al.*, 2013).

The crystal structure is stabilized by the intermolecular C—H···O, C—H···N hydrogen bonds (Table 1, Fig. 2), weak π-π stacking interactions [*Cg*2···*Cg*2 (2 - *x*, 2 - *y*, 2 - *z*) = 3.760 (2) Å] between the benzene rings of adjacent 3,4-dimethoxyphenyl groups and C—H···π contacts (Table 1).

### 2. Experimental

The title compound was prepared by our previously reported method (Mohamed *et al.*, 2013). Single crystals sutiable for X-ray difraction were grown up by the slow evaporation method from an ethanolic solution of (I). *M*.p. 424 K and 83% yield.

### 3. Refinement

H atoms were included in calculated positions and refined using a riding model with C—H = 0.95 - 0.99 Å and *U*_iso_(H) = 1.5*U*_eq_(*C*_methyl_) and = 1.2*U*_eq_(C) for other H atoms. One reflection (0 0 1) was affected by the beam stop and was omitted from the refinement.

### Crystal data

**Table:**

C_28_H_30_N_2_O_2_	*Z* = 2
*M**_r_* = 426.54	*F*(000) = 456
Triclinic, *P*1	*D*_x_ = 1.252 Mg m^−^^3^
Hall symbol: -P 1	Mo *K*α radiation, λ = 0.71073 Å
*a* = 8.900 (2) Å	Cell parameters from 856 reflections
*b* = 11.915 (3) Å	θ = 2.5–20.8°
*c* = 12.009 (3) Å	µ = 0.08 mm^−^^1^
α = 106.757 (6)°	*T* = 150 K
β = 96.007 (5)°	Block, colourless
γ = 108.170 (6)°	0.24 × 0.21 × 0.11 mm
*V* = 1131.8 (5) Å^3^	

### Data collection

**Table:**

Bruker APEX2000 CCD area-detector diffractometer	3953 independent reflections
Radiation source: fine-focus sealed tube	2219 reflections with *I* > 2σ(*I*)
Graphite monochromator	*R*_int_ = 0.086
φ and ω scans	θ_max_ = 25.0°, θ_min_ = 1.9°
Absorption correction: multi-scan (*SADABS*; Bruker, 2011)	*h* = −10→10
*T*_min_ = 0.668, *T*_max_ = 0.981	*k* = −13→14
8350 measured reflections	*l* = −14→14

### Refinement

**Table:**

Refinement on *F*^2^	Primary atom site location: structure-invariant direct methods
Least-squares matrix: full	Secondary atom site location: difference Fourier map
*R*[*F*^2^ > 2σ(*F*^2^)] = 0.058	Hydrogen site location: inferred from neighbouring sites
*wR*(*F*^2^) = 0.127	H-atom parameters constrained
*S* = 0.87	*w* = 1/[σ^2^(*F*_o_^2^) + (0.0391*P*)^2^] where *P* = (*F*_o_^2^ + 2*F*_c_^2^)/3
3953 reflections	(Δ/σ)_max_ < 0.001
292 parameters	Δρ_max_ = 0.25 e Å^−^^3^
0 restraints	Δρ_min_ = −0.25 e Å^−^^3^

### Special details

**Table:**

Geometry. Bond distances, angles *etc*. have been calculated using the rounded fractional coordinates. All su's are estimated from the variances of the (full) variance-covariance matrix. The cell e.s.d.'s are taken into account in the estimation of distances, angles and torsion angles
Refinement. Refinement on *F*^2^ for ALL reflections except those flagged by the user for potential systematic errors. Weighted *R*-factors *wR* and all goodnesses of fit *S* are based on *F*^2^, conventional *R*-factors *R* are based on *F*, with *F* set to zero for negative *F*^2^. The observed criterion of *F*^2^ > σ(*F*^2^) is used only for calculating -*R*-factor-obs *etc*. and is not relevant to the choice of reflections for refinement. *R*-factors based on *F*^2^ are statistically about twice as large as those based on *F*, and *R*-factors based on ALL data will be even larger.

### Fractional atomic coordinates and isotropic or equivalent isotropic displacement parameters (Å^2^)

**Table:**

	*x*	*y*	*z*	*U*_iso_*/*U*_eq_	
O1	1.0678 (2)	1.23924 (16)	1.28583 (16)	0.0391 (7)	
O2	1.0748 (2)	1.05208 (17)	1.34508 (16)	0.0404 (7)	
N1	0.5735 (2)	0.70894 (19)	0.89430 (18)	0.0259 (8)	
N2	0.7629 (2)	0.66063 (19)	0.98101 (18)	0.0278 (8)	
C1	0.7160 (3)	0.7530 (2)	0.9748 (2)	0.0262 (9)	
C2	0.6472 (3)	0.5534 (2)	0.9023 (2)	0.0257 (9)	
C3	0.5296 (3)	0.5811 (2)	0.8472 (2)	0.0272 (9)	
C4	0.8031 (3)	0.8829 (2)	1.0501 (2)	0.0268 (9)	
C5	0.8063 (3)	0.9857 (2)	1.0185 (2)	0.0305 (9)	
C6	0.8932 (3)	1.1059 (2)	1.0950 (2)	0.0316 (10)	
C7	0.9794 (3)	1.1250 (2)	1.2033 (2)	0.0287 (9)	
C8	0.9822 (3)	1.0229 (2)	1.2361 (2)	0.0291 (9)	
C9	0.8945 (3)	0.9045 (2)	1.1609 (2)	0.0282 (9)	
C10	0.4779 (3)	0.7773 (2)	0.8591 (2)	0.0302 (9)	
C11	0.5026 (3)	0.7972 (3)	0.7431 (2)	0.0338 (10)	
C12	0.3887 (4)	0.8550 (3)	0.7021 (2)	0.0409 (11)	
C13	0.4153 (4)	0.8893 (3)	0.5935 (3)	0.0567 (12)	
C14	0.3796 (5)	0.7783 (3)	0.4835 (3)	0.0731 (16)	
C15	0.6611 (3)	0.4312 (2)	0.8838 (2)	0.0269 (9)	
C16	0.8124 (3)	0.4217 (2)	0.9029 (2)	0.0306 (9)	
C17	0.8291 (3)	0.3083 (3)	0.8889 (2)	0.0348 (10)	
C18	0.6946 (4)	0.2014 (3)	0.8543 (2)	0.0355 (10)	
C19	0.5440 (3)	0.2083 (3)	0.8336 (2)	0.0360 (10)	
C20	0.5276 (3)	0.3225 (2)	0.8490 (2)	0.0316 (10)	
C21	0.3856 (3)	0.5008 (2)	0.7528 (2)	0.0285 (9)	
C22	0.4042 (3)	0.4467 (3)	0.6400 (2)	0.0371 (10)	
C23	0.2726 (4)	0.3681 (3)	0.5509 (3)	0.0432 (11)	
C24	0.1200 (4)	0.3430 (3)	0.5736 (3)	0.0392 (11)	
C25	0.0999 (3)	0.3960 (3)	0.6849 (2)	0.0370 (10)	
C26	0.2314 (3)	0.4741 (2)	0.7744 (2)	0.0330 (10)	
C27	1.0564 (3)	1.3462 (2)	1.2624 (2)	0.0410 (11)	
C28	1.0836 (4)	0.9506 (3)	1.3800 (2)	0.0448 (11)	
H5	0.74750	0.97340	0.94260	0.0370*	
H6	0.89280	1.17570	1.07190	0.0380*	
H9	0.89540	0.83510	1.18430	0.0340*	
H10A	0.50770	0.86000	0.92220	0.0360*	
H10B	0.36180	0.73020	0.85150	0.0360*	
H11A	0.48440	0.71550	0.68170	0.0410*	
H11B	0.61570	0.85280	0.75310	0.0410*	
H12A	0.27640	0.79520	0.68590	0.0490*	
H12B	0.39940	0.93160	0.76790	0.0490*	
H13A	0.52930	0.94520	0.60760	0.0680*	
H13B	0.34560	0.93680	0.58040	0.0680*	
H14A	0.45340	0.73410	0.49350	0.1100*	
H14B	0.39440	0.80680	0.41520	0.1100*	
H14C	0.26760	0.72140	0.46970	0.1100*	
H16	0.90650	0.49540	0.92620	0.0370*	
H17	0.93380	0.30400	0.90320	0.0420*	
H18	0.70560	0.12260	0.84460	0.0430*	
H19	0.45050	0.13390	0.80840	0.0430*	
H20	0.42250	0.32630	0.83550	0.0380*	
H22	0.50970	0.46410	0.62370	0.0450*	
H23	0.28690	0.33090	0.47340	0.0520*	
H24	0.02840	0.28870	0.51160	0.0470*	
H25	−0.00590	0.37880	0.70060	0.0440*	
H26	0.21640	0.51020	0.85200	0.0400*	
H27A	1.09610	1.35060	1.19010	0.0610*	
H27B	1.12200	1.42190	1.32970	0.0610*	
H27C	0.94320	1.34030	1.25110	0.0610*	
H28A	0.97510	0.89910	1.38300	0.0670*	
H28B	1.15480	0.98290	1.45900	0.0670*	
H28C	1.12690	0.89930	1.32240	0.0670*	

### Atomic displacement parameters (Å^2^)

**Table:**

	*U*^11^	*U*^22^	*U*^33^	*U*^12^	*U*^13^	*U*^23^
O1	0.0477 (13)	0.0262 (12)	0.0363 (12)	0.0116 (10)	−0.0006 (10)	0.0057 (10)
O2	0.0492 (13)	0.0345 (12)	0.0327 (12)	0.0150 (10)	−0.0060 (10)	0.0095 (10)
N1	0.0273 (13)	0.0266 (13)	0.0258 (13)	0.0145 (11)	0.0038 (10)	0.0073 (10)
N2	0.0298 (13)	0.0273 (13)	0.0285 (13)	0.0127 (11)	0.0052 (11)	0.0102 (11)
C1	0.0301 (16)	0.0270 (16)	0.0233 (15)	0.0122 (13)	0.0046 (13)	0.0094 (13)
C2	0.0266 (15)	0.0259 (15)	0.0257 (15)	0.0119 (13)	0.0053 (12)	0.0079 (13)
C3	0.0313 (16)	0.0249 (16)	0.0255 (15)	0.0129 (13)	0.0068 (13)	0.0054 (13)
C4	0.0268 (15)	0.0273 (16)	0.0264 (16)	0.0130 (13)	0.0065 (12)	0.0056 (13)
C5	0.0304 (16)	0.0346 (17)	0.0267 (16)	0.0136 (14)	0.0020 (13)	0.0100 (14)
C6	0.0310 (16)	0.0291 (17)	0.0357 (17)	0.0119 (14)	0.0064 (14)	0.0114 (14)
C7	0.0278 (16)	0.0226 (16)	0.0303 (16)	0.0092 (13)	0.0040 (13)	0.0017 (13)
C8	0.0308 (16)	0.0304 (17)	0.0243 (15)	0.0138 (14)	0.0023 (13)	0.0047 (13)
C9	0.0304 (16)	0.0282 (16)	0.0295 (16)	0.0140 (13)	0.0081 (13)	0.0105 (13)
C10	0.0317 (16)	0.0296 (16)	0.0332 (16)	0.0178 (13)	0.0050 (13)	0.0096 (13)
C11	0.0352 (17)	0.0371 (17)	0.0342 (17)	0.0185 (14)	0.0072 (14)	0.0137 (14)
C12	0.053 (2)	0.0359 (18)	0.0388 (18)	0.0242 (16)	0.0055 (15)	0.0127 (15)
C13	0.072 (2)	0.062 (2)	0.049 (2)	0.032 (2)	0.0108 (19)	0.0298 (19)
C14	0.097 (3)	0.094 (3)	0.046 (2)	0.054 (3)	0.018 (2)	0.027 (2)
C15	0.0303 (16)	0.0279 (16)	0.0258 (15)	0.0145 (13)	0.0057 (13)	0.0096 (13)
C16	0.0292 (16)	0.0297 (16)	0.0320 (16)	0.0125 (13)	0.0016 (13)	0.0088 (13)
C17	0.0357 (18)	0.0369 (18)	0.0384 (18)	0.0208 (15)	0.0080 (14)	0.0141 (15)
C18	0.0472 (19)	0.0318 (17)	0.0375 (18)	0.0230 (16)	0.0131 (15)	0.0154 (14)
C19	0.0410 (19)	0.0280 (17)	0.0423 (18)	0.0120 (14)	0.0129 (15)	0.0159 (14)
C20	0.0297 (16)	0.0314 (17)	0.0378 (17)	0.0139 (14)	0.0096 (14)	0.0137 (14)
C21	0.0329 (16)	0.0265 (16)	0.0291 (16)	0.0163 (13)	0.0038 (13)	0.0086 (13)
C22	0.0347 (17)	0.0388 (18)	0.0362 (18)	0.0152 (15)	0.0061 (15)	0.0086 (15)
C23	0.043 (2)	0.049 (2)	0.0300 (18)	0.0180 (17)	0.0035 (15)	0.0021 (15)
C24	0.0372 (19)	0.0351 (18)	0.0375 (19)	0.0095 (15)	−0.0021 (15)	0.0083 (15)
C25	0.0298 (17)	0.0424 (19)	0.0365 (18)	0.0100 (15)	0.0039 (14)	0.0143 (15)
C26	0.0339 (17)	0.0326 (17)	0.0321 (16)	0.0131 (14)	0.0059 (14)	0.0094 (14)
C27	0.050 (2)	0.0277 (17)	0.0433 (19)	0.0128 (15)	0.0063 (16)	0.0117 (15)
C28	0.060 (2)	0.046 (2)	0.0379 (18)	0.0316 (18)	0.0031 (16)	0.0171 (16)

### Geometric parameters (Å, º)

**Table:**

O1—C7	1.358 (3)	C24—C25	1.361 (4)
O1—C27	1.413 (3)	C25—C26	1.368 (4)
O2—C8	1.359 (3)	C5—H5	0.9500
O2—C28	1.410 (4)	C6—H6	0.9500
N1—C1	1.362 (3)	C9—H9	0.9500
N1—C3	1.371 (3)	C10—H10A	0.9900
N1—C10	1.457 (3)	C10—H10B	0.9900
N2—C1	1.311 (3)	C11—H11A	0.9900
N2—C2	1.366 (3)	C11—H11B	0.9900
C1—C4	1.453 (3)	C12—H12A	0.9900
C2—C3	1.356 (4)	C12—H12B	0.9900
C2—C15	1.455 (4)	C13—H13A	0.9900
C3—C21	1.466 (4)	C13—H13B	0.9900
C4—C5	1.376 (3)	C14—H14A	0.9800
C4—C9	1.394 (3)	C14—H14B	0.9800
C5—C6	1.377 (3)	C14—H14C	0.9800
C6—C7	1.358 (3)	C16—H16	0.9500
C7—C8	1.389 (3)	C17—H17	0.9500
C8—C9	1.359 (3)	C18—H18	0.9500
C10—C11	1.506 (3)	C19—H19	0.9500
C11—C12	1.507 (5)	C20—H20	0.9500
C12—C13	1.495 (4)	C22—H22	0.9500
C13—C14	1.496 (5)	C23—H23	0.9500
C15—C16	1.387 (4)	C24—H24	0.9500
C15—C20	1.374 (4)	C25—H25	0.9500
C16—C17	1.370 (4)	C26—H26	0.9500
C17—C18	1.367 (5)	C27—H27A	0.9800
C18—C19	1.370 (5)	C27—H27B	0.9800
C19—C20	1.375 (4)	C27—H27C	0.9800
C21—C22	1.374 (3)	C28—H28A	0.9800
C21—C26	1.379 (4)	C28—H28B	0.9800
C22—C23	1.367 (4)	C28—H28C	0.9800
C23—C24	1.371 (5)		
			
C7—O1—C27	117.52 (19)	C11—C10—H10B	109.00
C8—O2—C28	116.8 (2)	H10A—C10—H10B	108.00
C1—N1—C3	107.2 (2)	C10—C11—H11A	109.00
C1—N1—C10	129.6 (2)	C10—C11—H11B	109.00
C3—N1—C10	123.3 (2)	C12—C11—H11A	109.00
C1—N2—C2	106.1 (2)	C12—C11—H11B	109.00
N1—C1—N2	110.8 (2)	H11A—C11—H11B	108.00
N1—C1—C4	126.0 (2)	C11—C12—H12A	108.00
N2—C1—C4	123.1 (2)	C11—C12—H12B	108.00
N2—C2—C3	110.3 (2)	C13—C12—H12A	109.00
N2—C2—C15	121.4 (2)	C13—C12—H12B	109.00
C3—C2—C15	128.3 (2)	H12A—C12—H12B	108.00
N1—C3—C2	105.7 (2)	C12—C13—H13A	109.00
N1—C3—C21	123.1 (2)	C12—C13—H13B	109.00
C2—C3—C21	131.1 (2)	C14—C13—H13A	109.00
C1—C4—C5	124.9 (2)	C14—C13—H13B	109.00
C1—C4—C9	117.4 (2)	H13A—C13—H13B	108.00
C5—C4—C9	117.7 (2)	C13—C14—H14A	109.00
C4—C5—C6	121.1 (2)	C13—C14—H14B	109.00
C5—C6—C7	120.2 (2)	C13—C14—H14C	110.00
O1—C7—C6	125.0 (2)	H14A—C14—H14B	109.00
O1—C7—C8	115.1 (2)	H14A—C14—H14C	109.00
C6—C7—C8	119.9 (2)	H14B—C14—H14C	109.00
O2—C8—C7	115.3 (2)	C15—C16—H16	119.00
O2—C8—C9	125.1 (2)	C17—C16—H16	119.00
C7—C8—C9	119.7 (2)	C16—C17—H17	120.00
C4—C9—C8	121.3 (2)	C18—C17—H17	120.00
N1—C10—C11	112.6 (2)	C17—C18—H18	120.00
C10—C11—C12	111.4 (2)	C19—C18—H18	120.00
C11—C12—C13	115.0 (3)	C18—C19—H19	120.00
C12—C13—C14	113.5 (3)	C20—C19—H19	120.00
C2—C15—C16	120.2 (2)	C15—C20—H20	120.00
C2—C15—C20	122.1 (2)	C19—C20—H20	120.00
C16—C15—C20	117.8 (2)	C21—C22—H22	120.00
C15—C16—C17	121.5 (2)	C23—C22—H22	120.00
C16—C17—C18	119.8 (3)	C22—C23—H23	120.00
C17—C18—C19	119.8 (3)	C24—C23—H23	120.00
C18—C19—C20	120.3 (3)	C23—C24—H24	120.00
C15—C20—C19	120.9 (3)	C25—C24—H24	120.00
C3—C21—C22	119.3 (2)	C24—C25—H25	120.00
C3—C21—C26	122.0 (2)	C26—C25—H25	120.00
C22—C21—C26	118.6 (2)	C21—C26—H26	120.00
C21—C22—C23	120.7 (3)	C25—C26—H26	120.00
C22—C23—C24	120.0 (3)	O1—C27—H27A	109.00
C23—C24—C25	119.9 (3)	O1—C27—H27B	109.00
C24—C25—C26	120.3 (3)	O1—C27—H27C	110.00
C21—C26—C25	120.5 (2)	H27A—C27—H27B	109.00
C4—C5—H5	119.00	H27A—C27—H27C	109.00
C6—C5—H5	120.00	H27B—C27—H27C	109.00
C5—C6—H6	120.00	O2—C28—H28A	109.00
C7—C6—H6	120.00	O2—C28—H28B	109.00
C4—C9—H9	119.00	O2—C28—H28C	109.00
C8—C9—H9	119.00	H28A—C28—H28B	109.00
N1—C10—H10A	109.00	H28A—C28—H28C	109.00
N1—C10—H10B	109.00	H28B—C28—H28C	110.00
C11—C10—H10A	109.00		
			
C27—O1—C7—C6	6.7 (4)	C2—C3—C21—C26	109.2 (3)
C27—O1—C7—C8	−173.8 (2)	C1—C4—C5—C6	−179.5 (3)
C28—O2—C8—C7	−178.5 (3)	C9—C4—C5—C6	−1.8 (4)
C28—O2—C8—C9	2.2 (4)	C1—C4—C9—C8	178.7 (3)
C3—N1—C1—N2	−0.3 (3)	C5—C4—C9—C8	0.9 (4)
C3—N1—C1—C4	−177.0 (2)	C4—C5—C6—C7	0.8 (4)
C10—N1—C1—N2	179.9 (2)	C5—C6—C7—O1	−179.3 (3)
C10—N1—C1—C4	3.2 (4)	C5—C6—C7—C8	1.2 (4)
C1—N1—C3—C2	0.5 (3)	O1—C7—C8—O2	−1.0 (3)
C1—N1—C3—C21	−177.1 (2)	O1—C7—C8—C9	178.4 (2)
C10—N1—C3—C2	−179.6 (2)	C6—C7—C8—O2	178.6 (2)
C10—N1—C3—C21	2.8 (4)	C6—C7—C8—C9	−2.0 (4)
C1—N1—C10—C11	100.4 (3)	O2—C8—C9—C4	−179.7 (3)
C3—N1—C10—C11	−79.4 (3)	C7—C8—C9—C4	1.0 (4)
C2—N2—C1—N1	−0.1 (3)	N1—C10—C11—C12	173.6 (2)
C2—N2—C1—C4	176.7 (2)	C10—C11—C12—C13	174.5 (3)
C1—N2—C2—C3	0.5 (3)	C11—C12—C13—C14	66.4 (4)
C1—N2—C2—C15	178.9 (2)	C2—C15—C16—C17	178.5 (2)
N1—C1—C4—C5	−33.0 (4)	C20—C15—C16—C17	−0.7 (3)
N1—C1—C4—C9	149.3 (3)	C2—C15—C20—C19	−179.2 (2)
N2—C1—C4—C5	150.7 (3)	C16—C15—C20—C19	−0.1 (3)
N2—C1—C4—C9	−27.0 (4)	C15—C16—C17—C18	0.6 (4)
N2—C2—C3—N1	−0.6 (3)	C16—C17—C18—C19	0.1 (4)
N2—C2—C3—C21	176.7 (2)	C17—C18—C19—C20	−0.9 (4)
C15—C2—C3—N1	−178.9 (2)	C18—C19—C20—C15	0.8 (4)
C15—C2—C3—C21	−1.5 (5)	C3—C21—C22—C23	177.9 (3)
N2—C2—C15—C16	−27.4 (3)	C26—C21—C22—C23	0.1 (5)
N2—C2—C15—C20	151.7 (2)	C3—C21—C26—C25	−178.2 (3)
C3—C2—C15—C16	150.8 (3)	C22—C21—C26—C25	−0.5 (4)
C3—C2—C15—C20	−30.2 (4)	C21—C22—C23—C24	0.3 (5)
N1—C3—C21—C22	108.4 (3)	C22—C23—C24—C25	−0.3 (5)
N1—C3—C21—C26	−73.9 (3)	C23—C24—C25—C26	−0.1 (5)
C2—C3—C21—C22	−68.5 (4)	C24—C25—C26—C21	0.5 (5)

### Hydrogen-bond geometry (Å, º)

Cg1 is the centroid of the N1/N2/C1–C3 imidazole ring.

**Table:**

*D*—H···*A*	*D*—H	H···*A*	*D*···*A*	*D*—H···*A*
C23—H23···O1^i^	0.95	2.53	3.168 (4)	125
C27—H27*A*···N2^ii^	0.98	2.51	3.474 (3)	168
C27—H27*A*···*Cg*1^ii^	0.98	2.77	3.608 (3)	144

Symmetry codes: (i) *x*−1, *y*−1, *z*−1; (ii) −*x*+2, −*y*+2, −*z*+2.
